# Diseases of the Rectum Stimulating Uterine Disease

**Published:** 1872-05

**Authors:** 


### SELECTIONS.

### DISEASES OF THE RECTUM SIMULATING UTERINE DISEASE.

Dr. Edis, of the Hospital for Women, Soho Square, London, reported the following cases to the *Medical Times and Gazette*, November 4, 1871:

Case 1.—G. R , aged 25; married seven years; resides in India; sterile. For years passed she has suffered from intense pain in sacral region; worse on exertion and defæcation. The pericds were regular, scanty, and painful. After being under medical care for several years in India, her husband sent her over to England to consult the doctors here, and see if any benefit could be obtained. At the time of my seeing her, in June, 1869, five weeks before her return to India, she had been under the care of several medical men, had had the cervix divided, had worn an intra-uterine stem, as also a Hodge's pessary; had been leeched and blistered, and had evidently gone through the usual routine treatment, without any alleviation of her sufferings. She now despaired of ever getting better, and was very miserable at the thought of having to return to India after a twelvemonth's absence, having spent a little fortune on doctors, and derived little or no benefit as regards the pain in sacrum, although the dysmenorrhœa was certainly less, and her general health improved. On examination, she complained of much pain the moment the finger entered the vagina, which induced me to examine carefully the rectum, and there I discovered an ulcer the size of a shilling on the posteria wall, just above the sphincter, which was exposed, and very painful; the margin was indurated, and the ulcer was evidently of long standing. Having given a small dose of calomel to clear the portal system, and instructed her to use an enema, I divided the ulcer through its centre on either side, confined her to bed, gave opium supositories, restricted her to spoon di[illegible]t, and at the end of a week induced the bowels to act by castor [illegible]l. A solution of nitrate of silver was applied once to stimul[illegible]e the surface, and some pills consisting of pil. hyd. ipec. and rh[illegible]i. co. were ordered, to secure the regular action of the bowels, which for many years had been very costive.

September, 1871.—Patient has just returned from India, and called to report herself, and tell me how she had been, having had no return of the disease.

Case 2.—L. M., aged 37; married twelve years; sterile. Had been doctoring for fifteen months before my seeing her in March, 1871. Complained of vaginal discharge, which caused excoriation of the vulva and anal cleft; dreadful aching pain in groin, burning and smarting at times; aching pain in loins and lower back, "as if it opened and shut." Could not walk a hundred yards without wanting to pass water, or feeling so weak that she was obliged to sit down; had constant desire to go to the closet, especially on first rising of a morning, and after eating or drinking anything, when she noticed blood and slime with the motion, and on some occcasions she had passed large quantities of blood. Coitus was so painful she dreaded the idea of it. She felt so miserable her life was a burden to her. She got little sympathy from her medical men, who, after leeching and lotioning the uterus, and pouring in no end of tonics, failed to afford her any relief.

On examination, a long fissure extended up the anal cleft, and the skin generally was dry and irritable. The lower portion of the rectum was extensively ulcerated and intensely painful, thick, slimy mucus, tinged with blood, covering the surface. An ethereal solution of arg. nit. scr. ij. ad. dr. j. was applied pretty freely externally. Liq. hyd. nitrat. acid. was carefully applied to several of the ulcerated points, and an enema of glyc. acid. gallici with tinct. bellad. in lotio plumbi was ordered to be used night and morning; the bowels kept gently open with conf. sennæ, and a mixture of ferri and mag. sulph. with liquor strichniæ administered thrice daily. Within a month from this date she was nearly well. The mixture persevered in for a few weeks longer, when she was discharged convalescent.

I saw her on October 24, as she had a slight return of the external fissure; but with this exception she had remained perfectly well, and has had no return of the distressing symptoms from which she suffered. Her general health is vastly improved, and the change in her mental condition is gratifying to witness; she is quite a different being.

Case 3.—M. B., aged 44; married twenty-two years; two children. Complains of constant aching pain in lower back and in right iliac region; frequent desire to micturate; feeling of pressure and bearing down, worse on walking; bowels generally confined; sense of weariness and fatigue; aching in upper part of thighs; irritation of the privates, and feeling of fulness as if they were swollen; has frequent spasms in lower abdomen. Has suffered from these symptoms for the last ten months, spite of all treatment, until she began to fear it must be cancer, and came up 150 miles to consult me.

On examination, the uterus was found to be bulky, but mobile; not tender on pressure, and not misplaced. The pain was principally referred to the right iliac region; the cæcum seemed to be much distended, and was apparently blocked up with fæces. A mixture of nitro-muriatic acid, quinine and sp. chlor., and pills of extract of belladonna, with aloes and rhubarb, were ordered with a confectio sennæ if necessary. She returned home, and after persevering with the treatment for a month, wrote to inform me that she had quite lost all her symptoms, and was very much improved in health.

*Remarks.*—This seems to have been one of those cases frequently met with in females, of pain in the cæcum, caused by over-distension either from wind, or fæces, due entirely to constipation. It is here that belladonna acts so beneficially in relieving the spasm, and insuring regular action of the bowels.

